# High Variability in the Use of Cement for Femoral Stem Fixation in Hip Fractures—An Analysis of the Canadian Joint Replacement Registry

**DOI:** 10.3390/jcm14155463

**Published:** 2025-08-04

**Authors:** Fernando Diaz Dilernia, Eric Bohm, Gavin C. A. Wood

**Affiliations:** 1 Adult Hip and Knee Reconstructive Surgery, Division of Orthopaedic Surgery, Department of Surgery, Queen’s University & Kingston Health Sciences Centre, Kingston, ON K7L 2V7, Canada; gavin.wood@kingstonhsc.ca; 2 Concordia Hip & Knee Institute, Concordia Joint Replacement Group, Suite 310-1155 Concordia Ave, Winnipeg, MB R2K 2M9, Canada; ebohm@cjrg.ca

**Keywords:** hip fractures, femoral stem fixation, cemented, uncemented, joint registries

## Abstract

**Background**: This study examines current trends in Canada using data from the Canadian Joint Replacement Registry (CJRR) and includes a national survey to understand the varied uptake of cement for femoral stem fixation. **Methods**: The survey was available online and the website link was distributed to all orthopaedic surgeons through the Canadian Orthopaedic Association between September and December 2022. The CJRR obtained data from the Canadian Institute for Health Information (CIHI), and information pertaining to patients 55 years of age and older who underwent hemiarthroplasty for hip fracture in Canada between April 2017 and March 2022 was used. **Results**: Most respondents practiced in an academic community setting (52%). Only 53% of respondents reported using cement, and 71% indicated that cemented fixation was the best practice. The main reasons for using uncemented stems were less operative time (23%), cement disease concerns (11%), and surgeons’ comfort (10%). Similarly, CJRR data showed only 51% cemented fixation among 42,386 hemiarthroplasties performed between 2017 and 2022. The proportion of cemented implants varied by province, but overall, the increase in the use of cement from 2017 to 2022 was from 42.9% to 57.7%. **Conclusions**: This study demonstrates variability in the use of cement for femoral fixation despite solid evidence showing improved outcomes using cement. Some of the main reasons in favour of uncemented stems include operative time, surgical training, and concerns about cement disease. Establishing clear position statements and guidelines supporting cemented fixation may be prudent to build universal consensus on this practice.

## 1. Introduction

Hip fractures are associated with significant morbidity and mortality. Approximately one-quarter of patients who sustain such injuries will die within one year [1]. Between 2019 and 2020, the incidence of hip fractures in Canadians aged 40 years and above was 156 per 100,000 [2]. With an aging population, hip fractures pose significant costs to the Canadian healthcare system and are expected to pose a burden of $2.4 billion by the year 2041 [3].

The literature supports the use of cemented over non-cemented femoral stem fixation for enhanced clinical outcomes, including improved mobility, decreased pain, and a reduced risk of revision [4,5]. The British Orthopaedic Association (BOA) issued a position statement recommending cemented stems when total hip arthroplasty (THA) is performed for community-functioning individuals with hip fractures [6]. Similarly, the American Academy of Orthopaedic Surgeons (AAOS) issued a ‘strong recommendation’ for cemented femoral fixation in older adults within its most recent clinical guidelines [7]. These recommendations are based on high-quality evidence and broad expert consensus demonstrating superior outcomes with cemented stems. However, concerns regarding bone cement implantation syndrome (BCIS)—a rare but potentially serious complication—have influenced practice patterns. BCIS is characterized by hypoxia, hypotension, and cardiac instability during cementation [8,9].

The Canadian Joint Replacement Registry (CJRR) houses patient-specific information (clinical, surgical, and prosthesis) on hip and knee replacement surgeries performed in Canada, except for Quebec and the northern territories. Current reports from the CJRR indicate that patients treated with cemented hemiarthroplasty following hip fracture have a lower risk of revision compared to those treated with cementless fixation. When surgeon volume was examined, those using cemented stems continued to have lower revision rates over cementless stems whether the surgeon performed greater or less than 50 hip arthroplasties per year for acute femoral neck fractures [10]. Despite the evidence, the vast majority of surgeons still do not use a cemented fixation for hip arthroplasty in Canada [11]. Several reasons contribute to the underutilization of cemented implants in practice, including increased operative time, individual surgeon preferences, and “Cement disease” or “bone cement implantation syndrome” [8,9]. While an exact definition of bone cement implantation syndrome is yet to be agreed upon, it refers to a rare complication of cemented bone surgery characterized by hypoxia, hypotension, and loss of consciousness around the time of cementation [8,9].

Given the widespread variation in the use of cement for femoral stem fixation, our objective was to evaluate provincial trends in cement utilization using CJRR data and to assess Canadian orthopaedic surgeons’ perspectives via a national survey. We hypothesized that despite clinical guidelines recommending cementation, its use remains inconsistent across Canada due to surgeon preference, training, and perceptions of BCIS.

## 2. Methods

This project obtained approval from the Research Ethics Board. The authors developed all survey questions. The Canadian Arthroplasty Society (CAS) approved this national survey prior to its endorsement by the Canadian Orthopaedic Association (COA) for distribution to all its members.

The survey was hosted online through Qualtrics, and the link was distributed via email through the COA to orthopaedic surgeons across Canada. Weekly reminders were sent over a three-month period (September–December 2022). There were no incentives to participate, and survey responses remained anonymous. While the survey was not formally piloted or tested for reliability, questions were based on expert consensus.

The CJRR obtained data from the Canadian Institute for Health Information (CIHI) main hospitalization and ambulatory care databases, the Discharge Abstract Database (DAD), and the National Ambulatory Care Reporting System (NACRS). Information pertaining to patients 55 years of age and older who underwent hemiarthroplasty for hip fracture in Canada (excluding Quebec) between 1 April 2017 and 31 March 2022 was used. Cemented or cementless fixation methods, as well as demographic and hospitalization characteristics, were included.

### Statistical Analysis

Continuous variables were expressed as means and ranges or medians and interquartile ranges (IQRs) depending on whether they had a normal distribution. Categorical variables were reported as frequencies and percentages. Continuous variables were compared using independent-sample *t*-tests when the data were normally distributed, and the Mann–Whitney U test was used otherwise. Categorical variables were compared using Chi-squared and Fisher’s exact tests. Variables were considered statistically significant at the *p*-value < 0.05. Data analyses were performed using IBM SPSS Statistics Version 29.0 (IBM Corp., Armonk, New York, NY, USA).

## 3. Results

The survey was distributed to 1600 individuals and members of the COA and received 106 responses, yielding a response rate of 7%. Respondents’ location of practice varied: Ontario was the most common (40%), followed by Quebec (12.3%) and British Columbia (9.4%). The mean and median number of years in practice as an orthopaedic surgeon were 17 and 14, respectively. Most respondents practiced in an academic setting (52%) as opposed to a community setting (48%). Detailed information on these demographics can be found in Table 1.

Most respondents participated in trauma calls, elective THA, and THA for hip fractures, which means that most of the participants were fellowship trained in arthroplasty and/or trauma. The number of respondents who reported using cement for all femoral stems in hip fracture repairs was roughly equal, with 53% responding ‘yes’ and 47% responding ‘no.’ These details regarding respondents’ surgical practice can be found in Table 2.

When asked about best practices for hip fracture care, 71% of respondents believed that cemented femoral fixation was the best practice, 27% indicated that both cemented and uncemented fixation were acceptable, and 2% thought that uncemented fixation was the best practice. With regards to the reason for the selection of an uncemented femoral stem over a cemented one, respondents cited less operative time (23%), concern about cement disease (11%), and individual comfort with performing uncemented stem fixation (10%). Respondents also indicated that a range of factors had the potential to increase their use of cemented stems, including auditing of their surgical practice, a position statement of national guidelines supported by the COA, and training in cemented surgical technique. When directly questioned whether an increase in the fee schedule for using cement would be an incentive to compensate for a perceived increase in operative time, few surgeons felt that this would be beneficial. Details of respondents’ knowledge, attitudes, and perceptions regarding cemented versus uncemented femoral stem fixation are presented in Table 3.

These results are comparable to the CJRR data, which examined hemiarthroplasties for hip fractures in Canada. They found that between 2017 and 2022, 42,386 hemiarthroplasties for hip fracture repair were used, and 51% of these implants were cemented. Furthermore, when examining trends by province, the proportion of cemented implants used for hip fractures in 2017 was lowest in Manitoba at 10% and highest in Prince Edward Island at 88%. Over time, there was an increase in cement use in most provinces, the greatest from 10% to 30% in Manitoba. Overall, the increase in cement use from 2017 to 2022 across all provinces was from 42.9% to 57.7%.

## 4. Discussion

Unfortunately, the survey response rate was only 7%, a limitation that significantly impacts the generalizability of our findings. This low participation introduces the risk of reporter bias and should be considered when interpreting the survey results.

**Figure 1:**
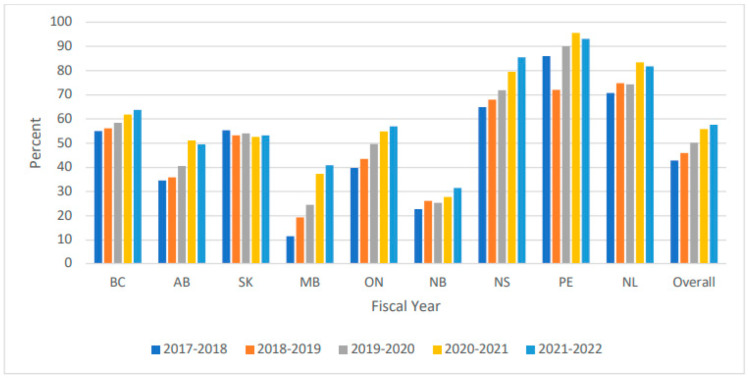
Percentage use of cement hemiarthroplasty in hip fracture fixation by province. Discharge Abstract Database and National Ambulatory Care Reporting System, 2017–2018 to 2021–2022, Canadian Institute for Health Information.

However, this cross-Canada survey reveals considerable variation in the use of cement, with 53% of respondents indicating that they used cement in all their hip fracture repairs and 47% indicating that they did not. This result is consistent with other Canadian studies reporting the use of cement for hip fracture repair. A retrospective study based in Nova Scotia noted that, while the rates of cement use have increased over recent years, its use remains relatively low. This study found that, from a sample of 3787 patients undergoing arthroplasty for hip fracture, 58.6% of patients received cemented implants [11]. Information from the CJRR reports a similar uptake of cemented stems, with 56.9% of hip fixation surgeries using cemented stems between 2020 and 2021, against a background of considerable provincial variation [10]. Although the number of respondents to this current survey was low, it would appear to reflect the orthopaedic forum in Canada, as the results mirror those from the CJRR (Figure 1).

It is worthwhile to consider solutions that may reduce the variability currently seen in cement use for hip fracture treatment. Some potential strategies include a formal position statement by the COA or national clinical guidelines recommending cemented femoral stem fixation. Since this survey was undertaken, the Choosing Wisely campaign, under the leadership of the COA, has published new practice recommendations in February 2023. One recommendation is to refrain from using cementless stem fixation when performing arthroplasty for femoral neck fractures in elderly patients due to the associated increased risk of periprosthetic fracture, increased risk of revision, and lack of reduction in mortality risk [12].

Another recommended strategy is distributing personalized feedback to surgeons regarding their patients’ postoperative performance. Such an endeavour has been piloted in Manitoba, where a provincial standards committee produces customized reports on an annual basis to orthopaedic surgeons. These personalized reports include individual surgeons’ and peer group data for comparison. Outliers are identified, and a remedial educational program is undertaken if required. Some of the information collected includes patients’ demographics and surgical quality indicators, length of hospital stay, proportion of cemented fixations, transfusion rates, 30-day mortality rates, and re-operation rates. One result of using this level of surgeon-specific feedback was a 29% increase in cementation rates for hip fractures over four years. This demonstrates that a personalized feedback strategy may be highly effective in changing cement fixation practices if adopted across all provinces. Finally, given that 23% of respondents cited operative time as a barrier, implementing time-efficient cementation protocols and specific training may enhance adoption.

Our study has certain limitations that should be considered when interpreting our results. First, the evidence presented must be interpreted in the context of its methodology. A survey does not represent the specific treatment of a particular pathology but rather the general opinion on a clinical scenario. As with all perception-based surveys, responses are subject to biases in memory and judgment. Also, this study does not include all Canadian orthopaedic surgeons. Only a small fraction of surgeons at the national level responded, under-representing the response rate in community and academic centres, which may affect our results. Although our study includes national registry data, the CJRR does not include data from the province of Quebec, which comprises nearly one-third of the Canadian population. This omission may limit the generalizability of the registry findings at the national level. Additionally, the CJRR dataset used in this study does not include clinical outcomes such as mortality, age, comorbidities, or functional recovery, and multivariate analysis and outcome reporting were not performed due to the absence of these key confounding variables. On the other hand, the quality and completeness of DAD and NACRS have been very high, and it is unlikely that hip fractures were excluded, as recording is mandatory in all of Canada (except Quebec). This omission, particularly the exclusion of Quebec, which comprises approximately one-third of Canada’s population, may limit the national generalizability of these findings. However, to our knowledge, this is the first study to incorporate CJRR data and a survey in examining the controversial use of cemented fixations for hip fractures in Canada. Further longitudinal research is necessary to confirm and extend our findings, as well as to address these limitations.

## 5. Conclusions

This Canadian study demonstrates variability in the use of cement for femoral stem fixation despite solid evidence demonstrating improved clinical outcomes with a cemented approach. Some of the main reasons cited by respondents to our supplemental survey in favour of an uncemented approach to hip fracture surgery include operative time, confidence in performing each technique, and concern about cement disease. With this knowledge, institutional-level support for surgeons could take the form of focused training sessions on the evidence behind this surgical approach and the practical skills necessary to apply it. Such efforts aim to improve consistency in the use of cemented stem fixation by equipping trainees with the knowledge, skills, and confidence to apply this approach effectively. Finally, to build consensus on this practice, it may be prudent for Canadian professional associations and health quality committees to establish clear position statements and clinical practice guidelines supporting cemented femoral stem fixation.

## Figures and Tables

**Table 1:** Respondent Characteristics.

**Practice Location**	**Number (%)**
Ontario	42 (39.6%)
Quebec	13 (12.3%)
British Columbia	10 (9.4%)
Alberta	9 (8.5%)
Nova Scotia	5 (4.7%)
Saskatchewan	5 (4.7%)
Manitoba	3 (2.8%)
Newfoundland and Labrador	3 (2.8%)
New Brunswick	2 (1.9%)
Northwest Territories	1 (0.9%)
United States	4 (3.8%)
Non-North America	2 (1.9%)
Missing	7 (6.6%)
Total	106 (100%)
**Practice Type**	**Number (%)**
Academic	53 (52%)
Community	49 (48%)
**Years of Practice**	**Number**
Mean	17.3
Median	14
Minimum	1
Maximum	45

**Table 2:** Surgical Practice Characteristics.

**Do you do trauma call?**	**Percentage**
Yes	89%
No	11%
**Do you perform elective THA?**	**Percentage**
Yes	82%
No	18%
**Do you perform THA for hip fractures?**	**Percentage**
Yes	71%
No	29%
**Do you cement all your femoral stems for HA/THA for hip fracture repairs?**	**Percentage**
Yes	53%
No	47%
THA: total hip arthroplasty; HA: hemiarthroplasty	

**Table 3:** Respondents’ Knowledge, Attitudes, Perceptions.

**Characteristic**	**Percentage**				
**According to the evidence for best practice on hip fracture care, what is your understanding regarding femoral fixation choice?**					
Cemented	71%				
Uncemented	2%				
Both are acceptable	27%				
**Which of the following factors influence your decision to perform an uncemented stem instead of cemented stem for hip fracture repair (choose all that apply)?**					
Less operative time	23%				
Concern about cement disease	11%				
I am better at performing an uncemented stem fixation for hip fracture repair	10%				
Concern about fat emboli disease	8.5%				
Unfamiliar with cemented technique	5%				
**Please rank the following four factors according to how helpful they would be to increasing your use of cemented stems (1 = most important)**	**1**	**2**	**3**	**4**	**5**
Audit of your hip fracture performance	25%	31%	25%	10%	9%
COA position statement and national guidelines	12%	31%	28%	23%	6%
Training in the cement technique	23%	12%	15%	31%	19%
Premium fee code for the use of cement	14%	23%	16%	31%	16%
N/A—I use cement every time	72%	2%	3%	0%	23%

## Data Availability

The raw data supporting the conclusions of this article will be made available by the authors on request. The data from the registry is available online on the website of the CJRR.
